# Complete Genome Sequence of *emm*1 Streptococcus pyogenes 10-85, a Strain Isolated from a Patient with Streptococcal Toxic Shock Syndrome in Japan

**DOI:** 10.1128/MRA.00453-19

**Published:** 2019-06-13

**Authors:** Ichiro Tatsuno, Masanori Isaka, Masakado Matsumoto, Naomi Nishio, Hideyuki Matsui, Tadao Hasegawa

**Affiliations:** a Department of Bacteriology, Nagoya City University Graduate School of Medical Sciences, Nagoya, Japan; b Department of Microbiology and Medical Zoology, Aichi Prefectural Institute of Public Health, Nagoya, Japan; Broad Institute

## Abstract

Here, we announce the complete genome sequence of Streptococcus pyogenes strain 10-85 (type *emm*1), isolated from a patient with streptococcal toxic shock syndrome (STSS). The strain lacks the genomic regions encoding SalR-SalK, a two‐component regulatory system, and the adjacent type I restriction modification system.

## ANNOUNCEMENT

Streptococcus pyogenes (group A *Streptococcus* [GAS]) is a Gram-positive bacterium that infects the upper respiratory tract, including the tonsils and pharynx, and it is responsible for postinfection diseases, such as rheumatic fever and glomerulonephritis. S. pyogenes also causes severe invasive diseases, including necrotizing fasciitis and streptococcal toxic shock syndrome (STSS) (1–5). The virulence strength is considered to be dependent upon mutations in *covR*, *covS*, or *rgg*, negative regulators in *emm*1 clinical isolates, as reported previously (6–10).

Recently, we reported that the genomic regions encoding SalR-SalK, a two-component regulatory system, and the adjacent type I restriction modification system were deleted in some *emm*1-type isolates from both STSS and non-STSS patients in Japan. S. pyogenes strain 10-85 from an STSS patient is one of the isolates with the deletion (11), and it contained no mutations in *covR*, *covS*, and *rgg* (12). The strain 10-85 is resistant to macrolide and has a conjugative prophage Φ1207.3 (formerly Tn*1207.3*), which carries the macrolide resistance genes *mef*(A) and *msr*(D) (13, 14).

The S. pyogenes strain 10-85 genome has been previously sequenced, and a total of 27 contigs were obtained (12). To obtain the complete genome sequence, the strain 10-85 genome was resequenced on a PacBio RS II instrument (Pacific Biosciences, Menlo Park, CA) at TaKaRa Bio, Inc. (Shiga, Japan). The strain was cultured at 37°C in brain heart infusion (BHI) broth (E-MC62; Eiken Chemical Co., Tokyo, Japan) supplemented with 0.3% yeast extract (BD, Sparks, MD, USA) broth for 18 h without agitation. The cells collected by centrifugation were incubated at 37°C in 3.3 mg/ml achromopeptidase and 5 mM EDTA. After sodium dodecyl sulfate was added at 1.43% of the final concentration, the cells were further incubated for 10 min at 90°C. The genomic DNA was isolated by three freeze-thaw cycles and phenol-chloroform extraction. The genomic DNA was fragmented prior to PacBio RS II sequencing using the Covaris g-TUBE device (Woburn, MA), in accordance with the manufacturer’s instructions. PacBio RS II sequencing runs were performed using the PacBio SMRTbell template prep kit 1.0 and polymerase binding kit P6 after size selection using BluePippin (Sage Science, Beverly, MA) with a cutoff value of 15 kb. The high-quality filtered 105,098 subreads (with subread lengths of ≥500 bases, polymerase read lengths of ≥100 bases, and polymerase read qualities of ≥0.80) were assembled *de novo* using the Hierarchical Genome Assembly Process (HGAP) version 3 in the SMRT Analysis software version 2.3.0 (Pacific Biosciences), and a single contig was obtained. The overlap was removed to circularize the contig. Annotation was performed using the DDBJ Fast Annotation and Submission Tool (15).

The S. pyogenes strain 10-85 harbored a single circular genome of 1,778,006 bp, with an average G+C content of 38.6%. We observed 1,664 protein-coding regions, 18 rRNA operons, and 67 tRNA genes. The gene content matched the previously reported results (11–14).

### Data availability.

The whole-genome sequence of S. pyogenes strain 10-85 was submitted to DDBJ/ENA/GenBank under the accession number AP019548 and BioProject accession number PRJDB4033. The version described in this paper is the first version, AP019548.1. The accession number for the PacBio data in DDBJ/ENA/NCBI is DRA008374.
